# Enhanced therapeutic effects of MSC-derived extracellular vesicles with an injectable collagen matrix for experimental acute kidney injury treatment

**DOI:** 10.1186/s13287-020-01668-w

**Published:** 2020-04-22

**Authors:** Yue Liu, Jian Cui, Hongfen Wang, Kamal Hezam, Xiaotong Zhao, Haoyan Huang, Shang Chen, Zhibo Han, Zhong-Chao Han, Zhikun Guo, Zongjin Li

**Affiliations:** 1 School of Medicine, Nankai University, 94 Weijin Road, Tianjin, 300071 China; 2 The Key Laboratory of Bioactive Materials, Ministry of Education, Nankai University, the College of Life Science, Tianjin, 300071 China; 3 Department of Intensive Care Unit (ICU), People’s Hospital of Rizhao, Rizhao, 276826 Shandong China; 4 State Key Laboratory of Kidney Diseases, Chinese PLA General Hospital, Beijing, 102218 China; 5 Henan Key Laboratory of Medical Tissue Regeneration, Xinxiang Medical University, 601 Jinsui Road, Xinxiang, 453003 Henan China; 6 Jiangxi Engineering Research Center for Stem Cell, Shangrao, 334001 Jiangxi China; 7 Tianjin Key Laboratory of Engineering Technologies for Cell Pharmaceutical, National Engineering Research Center of Cell Products, AmCellGene Co., Ltd., Tianjin, China

**Keywords:** Mesenchymal stem cell (MSC), Extracellular vehicles (EVs), Collagen matrix, Molecular imaging, Acute kidney injury (AKI)

## Abstract

**Background:**

Mesenchymal stem cell (MSC)-derived extracellular vesicles (EVs) have been shown to have therapeutic potential for ischemic diseases and are considered an alternative to cell therapy. However, the low retention and poor stability of EVs post-transplantation in vivo remain obstacle prior to the clinical application of EVs.

**Methods:**

This study was designed to investigate whether collagen matrix could increase the retention and stability of EVs and further improve the therapeutic effects in murine acute kidney injury (AKI) model. EVs were isolated from human placental MSCs (hP-MSC-EVs) and encapsulated in a collagen matrix. Then, we investigated whether collagen matrix can prolong the retention of EVs in vivo, further enhancing the therapeutic efficiency of EVs in AKI.

**Results:**

Our results indicated that collagen matrix could effectively encapsulate EVs, significantly increase the stability of EVs, and promote the sustained release of EVs. Collagen matrix has improved the retention of EVs in the AKI model, which was proved by *Gaussia* luciferase (Gluc) imaging. The application of collagen matrix remarkably facilitated the proliferation of renal tubular epithelial cells in AKI compared with EVs alone. Moreover, collagen matrix could further augment the therapeutic effects of hP-MSC-EVs as revealed by angiogenesis, fibrosis and apoptosis, and functional analysis. Finally, we found that EVs play a therapeutic role by inhibiting endoplasmic reticulum (ER) stress.

**Conclusions:**

Collagen matrix markedly enhanced the retention of EVs and further augmented the therapeutic effects of EVs for AKI. This strategy for improving the efficacy of EVs therapy provides a new direction for cell-free therapy.

## Introduction

Acute kidney injury (AKI) is a syndrome characterized by a rapid decrease in renal excretion with the risk of developing chronic kidney disease (CKD) and ultimately leading to renal failure [1, 2]. Nevertheless, the kidney has relatively limited regenerative ability compared to other organs, such as the liver [2, 3]. Therefore, it is crucial for AKI therapy to regenerate the damaged kidney tissue and delay the progression of CKD to end stage renal disease (ESRD) [4–6]. In recent years, emerging evidences suggest that EVs isolated from several types of stem cells, such as mesenchymal stem cells (MSCs), embryonic stem cells (ESCs), endothelial progenitor cells, and induced pluripotent stem cells (iPSCs), exhibited renal protection and regeneration effects [4, 7–10]. Compared with stem cell therapy, EVs exhibit unique superiorities in safety issues. For instance, EVs derived from MSC have a low chance of eliciting immune responses, are highly stable, and are easy to store, thus avoiding the potential risk of ectopic transplantation [11, 12].

EV-based therapy has emerged as a promising strategy for restoring renal function followed by ischemia reperfusion injury. However, due to the short half-life of EVs in vivo and rapid clearance from the body after transplantation, the therapeutic application of EVs in kidney injury still faces challenges. Moreover, their functions may be compromised because the regeneration process usually takes a long time and the viability of free EVs cannot be maintained [13, 14]. Hence, the development of biocompatible gels capable of maintaining EV function and sustained release is essential for EV-based treatment. In addition, to monitor EVs in real time in vivo, it is urgent to establish an effective method for tracking EVs. Therefore, we introduced the EVs labeled with *Gaussia* luciferase (Gluc), which is unique to our laboratory, and used molecular imaging to achieve real-time monitoring in vivo. With the ability to visualize and to measure biological processes in intact animals instead of performing histological analysis at discrete time points, molecular imaging holds great potential for the study of EV-based therapies [11, 15].

Collagen is the most commonly used natural biomaterial in tissue engineering research. It has lots of biological activities, not only tissue support, but also an important component of extracellular matrix (ECM). When injected into the body, collagen could provide the niche that mimics the natural ECM to better exert the therapeutic effect [16, 17]. Previously, it has been reported that collagen matrix for tendon regeneration has good biocompatibility and mechanical properties, promoting cell adhesion and growth, and thereby behave as an ideal biomaterial for tissue regeneration [18]. Collagen matrix has a typical three-dimensional (3-D) cross-linking network with high drug/cell embedding rates and local maintenance of drugs/cells [19, 20]. Once encapsulated into the collagen matrix and injected into the tissues, cells, drugs, or EVs can be captured by the cross-linking network and released in a sustained manner [11, 21–25]. In addition, collagen matrix can provide a niche that mimics the natural ECM and promote cell adhesion, migration, and proliferation.

In this study, we hypothesized that the encapsulated EVs with thermosensitive, injectable collagen matrix will provide optimized effects on AKI. Therefore, we incorporated human placental MSC-derived EVs into collagen matrix. The degradable nature of collagen matrix allows the slow release of encapsulated EVs into surrounding tissues. Then, we investigated whether collagen matrix can prolong the retention of EVs in vivo, further enhancing the therapeutic effects of EVs after AKI. Our results indicated that collagen matrix-encapsulated EVs do have better therapeutic effects, suggesting that this is an effective approach to improve kidney regeneration.

## Materials and methods

### EV isolation

EVs were isolated as described previously [26, 27]. Human placenta-derived MSCs (hP-MSCs) were isolated as previously described and preserved in our lab [28]. The use of human-derived cells was approved by the Institutional Biomedical Research Ethics Committee of Nankai University School of Medicine. The culture supernatant was collected from hP-MSCs cultured in DMEM/F12 medium with EV-free FBS (Gibco) for 48 h and EVs were isolated by differential centrifugations as previously described [29]. In brief, the supernatant was centrifuged at 500*g* for 10 min at 4 °C to remove cells, at 2000*g* for 20 min to get rid of apoptotic bodies, and at 5000*g* for 30 min to remove cell debris. The resulting supernatant was centrifuged at 130,000*g* for 2 h at 4 °C in a SW32Ti rotor (Beckman Coulter, L-100XP Ultracentrifuge, CA). Then, the pellet was resuspended in PBS and subsequently ultracentrifuged at 130,000*g* again for 2 h to obtain purified EVs and stored at − 80 °C.

### Release kinetics of Col-EVs

The release rate of EVs was measured using an IVIS Lumina imaging system. In brief, 100 μg of Gluc-labeled EVs was encapsulated into 100 μL of collagen solution and gelatinized in a 48-well plate. Then, we immersed the Col-EVs with 200 μL of PBS and incubated in a 37 °C incubator. At the indicated time points, the supernatant was collected, and then transferred to another 48-well plate. Amount of EVs in the supernatant was determined by BLI analysis. The Gluc signal was detected as an equivalent of EVs. According to the trend of linear correlation between EVs content and Gluc activity, the Gluc signals were used to determine the EVs content [11].

### Animal model

We established an ischemic/reperfusion (IR) AKI model as previously described [8]. Briefly, male C57BL/6 Albino mice (8–10 weeks old, weighing 22–25 g) were anesthetized with 4% chloral hydrate (330 mg/kg) by intraperitoneal injection. The left renal pedicle was clamped with a nontraumatic microvascular clamp for 40 min after a flank dorsal longitudinal incision under sterile conditions, and the left kidney turned purple subsequent to clamping. Clamps were removed to initiate reperfusion, and the left kidney reverted to red within approximately 10 s. The body temperature was maintained with a homeothermic table during all procedures. After 5 min of reperfusion, approximately 100 μg of EVs suspended in PBS or collagen matrix gel was intrarenally injected into three sites of the left renal cortex (*n* = 10 per group). The final concentration of collagen was around 1.5 mg/ml. Equal injections of EVs, PBS, and collagen gel were used as controls. Sham-operated animals were subjected to a similar surgical procedure without IR injury or injection.

To monitor the in vivo angiogenic effects of EVs in real time, transgenic mice (Xenogen Corp, Hopkinton, MA) which express firefly luciferase under the promoter of vascular growth factor receptor2 (Vegfr2-luc) were used [2]. Protocols were approved by the Nankai University Animal Care and Use Committee guidelines which conform to the Guide for the Care and Use of Laboratory Animals published by the US National Institutes of Health (8th Edition, 2011).

### Bioluminescence imaging (BLI)

In the in vivo retention experiments, 100 μg of EVs labeled by Gluc were injected into the kidney cortex at a total volume of 30 μL. At different time points, we intraperitoneally injected Gluc’s substrate, coelenterazine (5 mg/kg; Nanolight Technology). The animals were imaged by BLI using the IVIS Lumina imaging system [11]. Furthermore, Fluc activity was used to real-time monitor the expression of the vegfr2 gene in vivo, as previously reported. The BLI signal was quantified by the average radiance from the region of interest (ROI) over the kidney after IRI injury [11, 30].

### Renal function analysis

Blood was harvested after severe AKI (unilateral ischemia/reperfusion and contralateral nephrectomy) via eyeball ablation and centrifuged at 3000 rpm for 10 min to obtain mouse serum. Blood urea nitrogen (BUN) and serum creatinine (SCr) levels were determined using a biochemical automatic analyzer (Vitalab Selectra E).

### Statistical analysis

All results presented are from at least three independent experiments for each condition. Data are expressed as means ± SEM. Statistical analysis was performed by one- or two-way ANOVA using the GraphPad Prism 5.0 software and the IBM SPSS STATISTICS 24. Differences were considered statistically significant at *P* < 0.05.

Detailed methods for EVs characterization and internalization, scratch wound healing, quantitative RT-PCR, western blot, preparation of collagen matrix, and histological analysis can be found in Supplemental Methods.

## Results

### Isolation and characterization of EVs

**Fig. 1:**
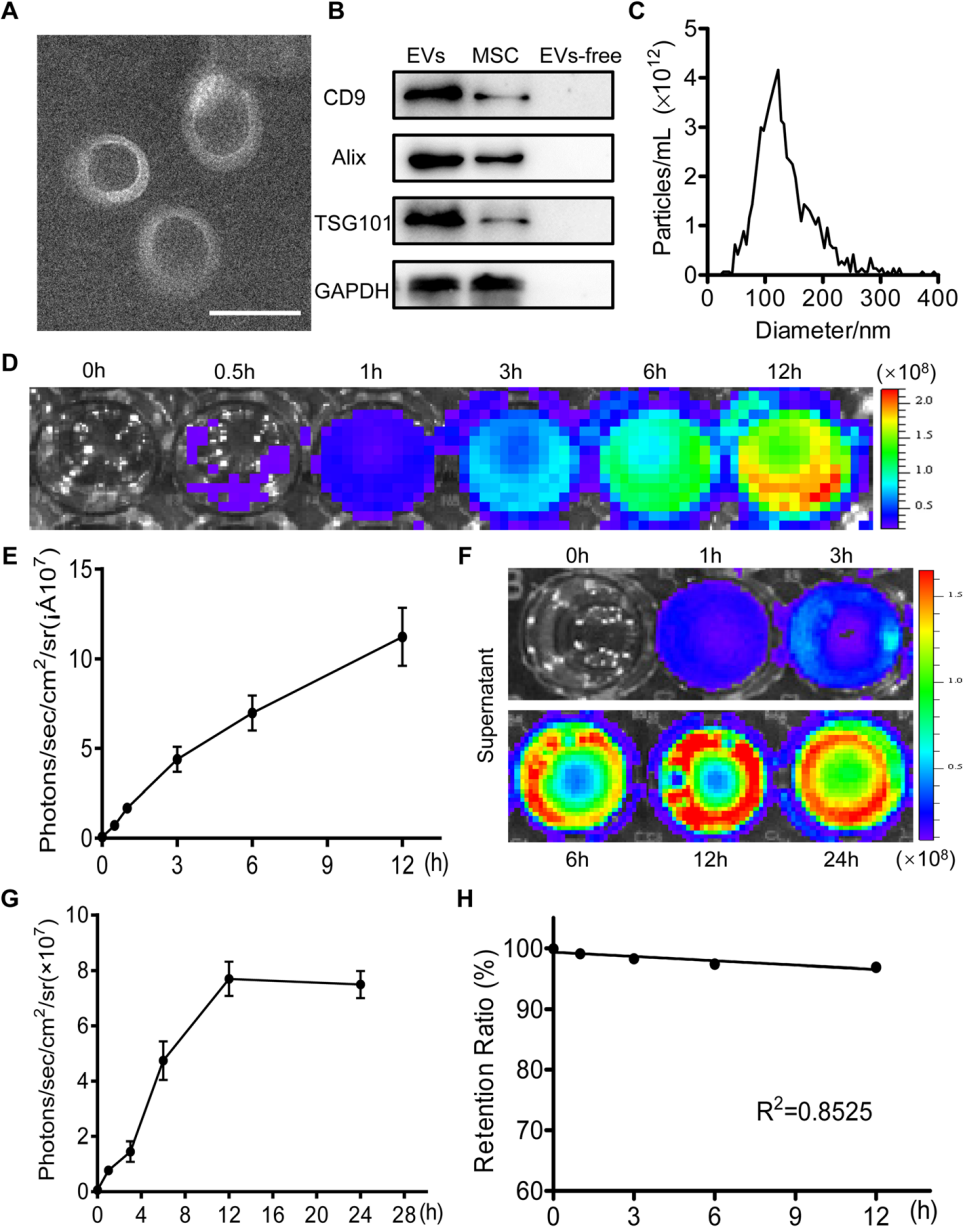
Characterization, internalization of EVs, and analysis of EV release from collagen matrix. **a** TEM image of EVs. Scale bar, 100 nm. **b** Western blot analysis of the marker of EVs, CD9, Alix, and TSG101 in EVs, MSCs, and EV-free conditioned medium. **c** Size distribution measure of EVs by NTA in scatter mode. **d**, **e** Internalization of Gluc-labeled EVs by HK-2 cells at various time points was analyzed with BLI. **f**, **g** The released EVs were determined by quantitative analysis of the Gluc signal. **h** Retention ratio of EVs in Col-EVs after immersing in PBS within 12 h (*R*^2^ = 0.8525). The signal activity was represented by photons/s/cm^2^/steradian

EVs were isolated from the supernatant of hP-MSCs via ultracentrifugation. Then, we characterized the EVs through TEM, western blotting, and NTA analysis. The TEM analysis showed that the EVs were round and cup-shaped vesicles of approximately 120 nm in diameter (Fig. 1a). Western blotting analysis results revealed that the expression levels of CD9, Alix, and TSG101 were significantly higher in the EVs compared with the MSCs, but no marker proteins were expressed in the supernatant after ultracentrifugation (Fig. 1b). Moreover, as shown in the nanoparticle tracking analysis (NTA), the average diameter of EVs was approximately 120 nm (Fig. 1c). These above data indicated the successful extraction of EVs from hP-MSCs.

### EVs internalization

For monitoring EVs in vivo real-time, we introduced Gluc-labeled EVs as reported before [11, 31]. BLI analysis revealed a significant linear correlation between the amount of EVs and Gluc activity (Figure S1). At the same time, the BLI images showed that the Gluc-labeled EVs were internalized by HK-2 cells and the Gluc signals increased over time (Fig. 1d, e). Furthermore, to further determine that EVs can be internalized by HK-2 cells, we labeled EVs with CM-DiI dye (red) and co-incubated with HK-2 cells expressing GFP (green) in vitro (Figure S2A, B, C). After incubation of HK-2 cells with DiI-labeled EVs for 12 h, we found that cells were able to uptake DiI-labeled EVs (Figure S2D). Taken together, these results proved that the bioluminescence reporter system and DiI dye were highly specific, reliable for labeling EVs, and EVs could be transferred into epithelial cells in vivo.

### Characterization of collagen matrix-encapsulated EVs in vitro and in vivo

The collagen matrix is a reticulated porous structure with high adhesion and has been applied in tissue repair post injures as an ideal delivery carrier for cells and drugs. The rat tail collagen I solution can form a three-dimensional architecture with a certain intensity at a concentration of 1 mg/mL or greater (pH = 7, 37 °C). The physical properties of the collagen matrix were examined by scanning electron microscopy (SEM) and rheological measurements. The results showed that the collagen matrix had an average mesh size of 10 μm, and the phase transition of the collagen solution to gel happened when the temperature rose to around 25 °C (Figure S3 A, B).

Based on the above collagen matrix, we have developed a novel strategy to improve the therapeutic effect of EVs. We encapsulated EVs into this injectable collagen matrix to form gel-incorporated EVs (Col-EVs), which could continuously release EVs to the surroundings. To further examine the release profile of the EVs, the concentration of EVs in the supernatant was determined by BLI analysis (Fig. 1f, g). Based on the trend of a linear correlation between the amount of EVs and Gluc activity (Figure S1), we could calculate the EVs concentration in the supernatant of PBS. Within 12 h, 0.24 μg of EVs were released per hour in the collagen matrix gel when 100 μg of Gluc-labeled EVs were encapsulated in the collagen matrix gel. Therefore, the retention ratio of EVs in the collagen matrix gel could be measured (Fig. 1h). These data suggested that collagen matrix could effectively encapsulate EVs, achieving sustained release in vitro.

**Fig. 2:**
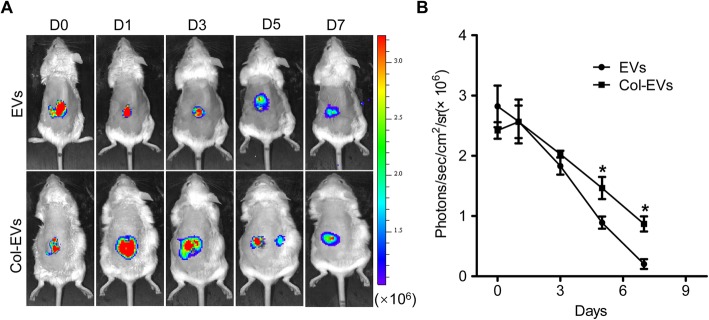
Collagen matrix enhanced the stability and retention of EVs. **a** The Gluc signals were tracked by BLI analysis to monitor the retention of the transplanted Col-EVs or EVs in vivo. **b** Quantitative analysis of Gluc signals demonstrated the retention of EVs in murine AKI model at all-time points. The signal activity was represented by photons/s/cm^2^/steradian. The concentration of substrate coelenterazine used in vivo was 5 mg/kg (*n* = 3; *P < 0.05 vs. EVs)

We hypothesized that collagen matrix gel could enhance the retention and stability of EVs in vivo. To validate this hypothesis, 100 μg of Gluc-labeled EVs mixed with collagen (Col-EVs) or suspended in PBS (EVs) were intrarenally injected into ischemic kidneys of mice at a 30 μL total volume. At the indicated time points, mice were imaged immediately after intraperitoneal injection of coelenterazine (5 mg/kg) using the IVIS Lumina Imaging System. Our BLI data exhibited the robust Gluc signals from the kidney region at day 1 in both groups, which indicated successful Gluc-EVs transplantation. However, the EVs group experienced severe donor EVs loss in the following time points compared to the Col-EVs group (Fig. 2). Overall, serial BLI imaging illustrated the enhanced retention of EVs incorporated in collagen matrix in vivo, indicating that collagen matrix might contribute to increase therapeutic potential of EVs.

### Enhanced proliferative effect of EVs in vitro and in vivo

**Fig. 3:**
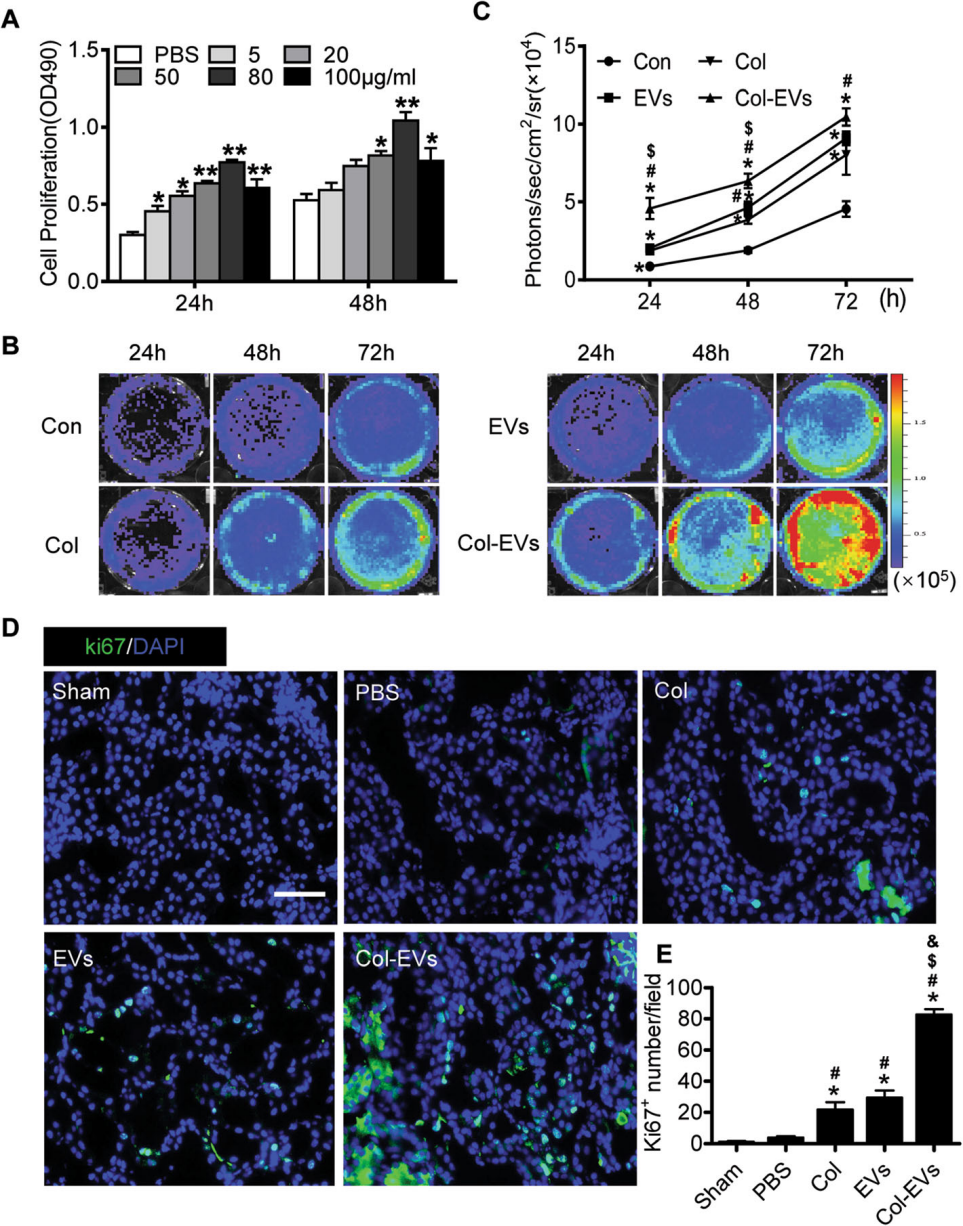
Collagen matrix accelerated proliferative ability of EVs. **a** The optimal EVs concentration for the highest viability of HK-2 cells is 80 μg/mL. Data were expressed as mean ± SEM. *P < 0.05, **P < 0.01 vs. PBS. **b** BLI assay revealed enhanced proliferation of HK-2 cells cultured with Col-EVs. **c** Quantitative analysis of BLI signals. The signal activity was expressed as photons/s/cm^2^/steradian. Data were expressed as mean ± SEM. *P < 0.05 vs. Con; ^#^*P* < 0.05 vs. Col; ^$^*P* < 0.05 vs. EVs. **d** Representative images showed the proliferation (Ki-67, green) of renal cells 3 days after AKI. Scale bar, 100 μm. **e** Quantification of the proliferation index of renal cells. Data were expressed as mean ± SEM. (*n* = 5; *P < 0.05 vs. sham; ^#^*P* < 0.05 vs. PBS; ^$^*P* < 0.05 vs. Col; ^&^*P* < 0.05 vs. EVs)

One of the characteristic pathological manifestations of AKI is renal tubular epithelial cell necrosis and loss [32] . Related works have proved that EV-based therapies have great potential for kidney regeneration and collagen has robust capacity to promote cell proliferation [33]. To analyze the optimal working concentration of EVs, HK-2 cells were cultured with EVs for 24 and 48 h. The CCK-8 assay revealed that EVs promoted HK-2 cell proliferation with an increased concentration at 24 and 48 h and the peak was 80 μg/mL (Fig. 3a). The scratch wound-healing assay was used to evaluate the pro-migratory effects of HK-2 cells. Col-EVs stimulation markedly increased cell migration (Figure S4A, B). BLI experiments showed that Col-EVs promoted HK-2 to expand more rapidly than EVs. Collagen treatment alone could significantly promote cell proliferation (Fig. 3b, c). We next investigated whether Col-EVs could promote cell proliferation by proliferating cell nuclear antigen (Ki-67) staining. The number of Ki-67^+^ cells of the Col group was markedly higher than that of the PBS group and further increased in the Col-EV-administrated group at day 3 (Fig. 3d, e and Figure S4C, D). Collectively, these data suggested that collagen matrix could improve the proliferative capacity of hP-MSC-derived EVs.

### Collagen matrix accelerated cytoprotective ability of EVs

**Fig. 4:**
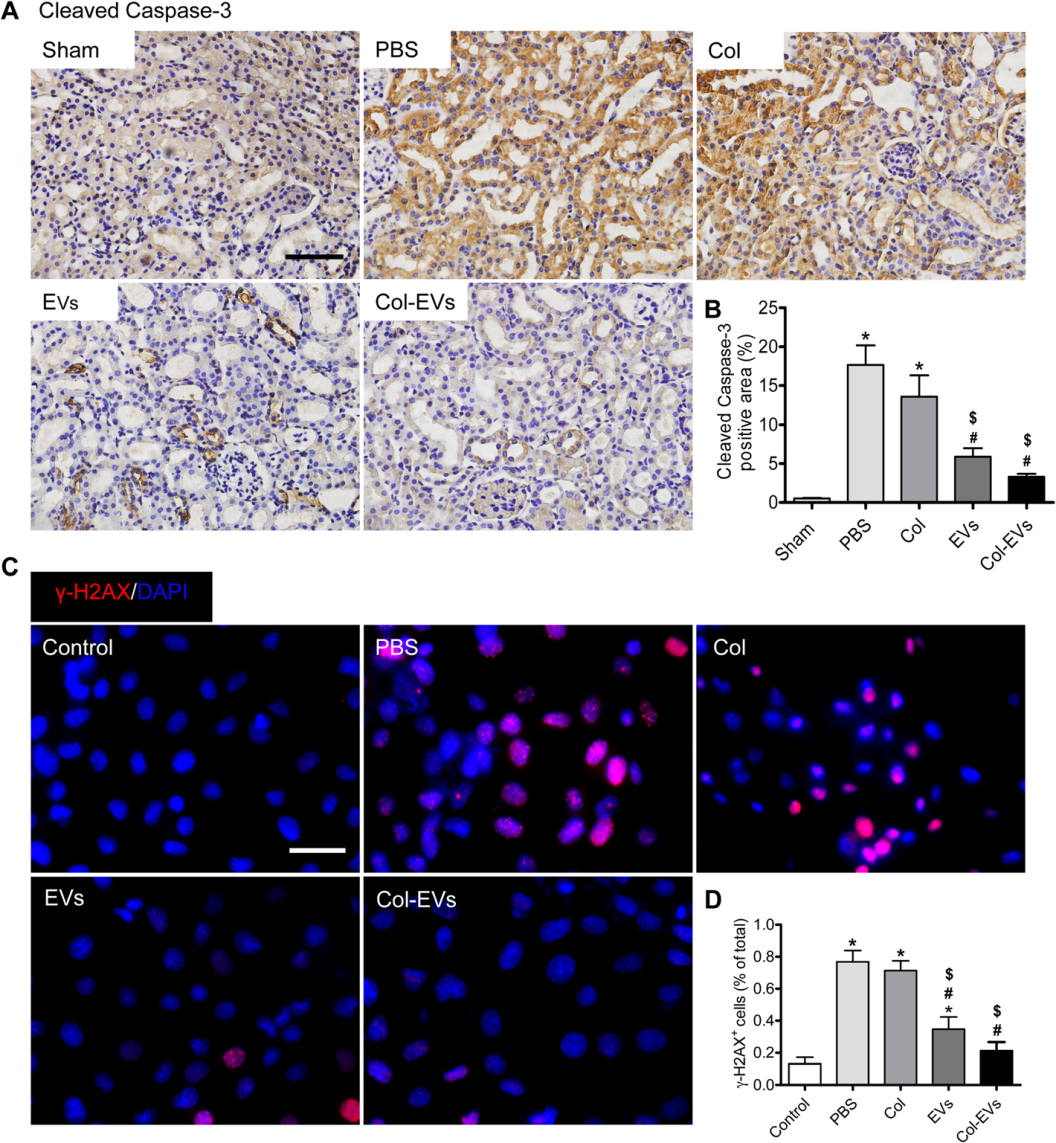
Col-EVs treatment alleviated apoptosis in vivo and in vitro. **a** Immunohistochemical staining of cleaved caspase-3 at day3 after AKI. Scale bar, 50 μm. **b** Quantification of cleaved caspase-3^+^ staining area in each group. Data were represented as mean ± SEM (*n* = 5; ^*^P < 0.05 vs. sham; ^#^*P* < 0.05 vs. PBS; ^$^*P* < 0.05 vs. Col). **c** Representative immunofluorescence images of γ-H2AX (red) expression in HK-2 cells. Scale bar, 50 μm. **d** Quantification of γ-H2AX^+^ cells in each group. Data were represented as mean ± SEM. *P < 0.05 vs. control; ^#^*P* < 0.05 vs. PBS; ^$^*P* < 0.05 vs. Col.

The apoptosis of renal tubular epithelial cells is partly responsible for IR-induced AKI. To assess the role of Col-EVs in protecting renal tubular epithelial cells from oxidative stress injury caused by ischemia reperfusion, we stained the kidney sections with cleaved caspase-3. The results showed that the expression of cleaved caspase-3 in the EVs and Col-EVs groups was greatly reduced compared with the PBS group (Fig. 4a, b). Additionally, we established a model of apoptosis induced by H_2_O_2_ in vitro and detected the DNA damage of cells by γ-H2AX immunofluorescence staining. The results indicated that Col-EVs treatment could significantly reduce DNA damage compared with other groups (Fig. 4c, d). All these findings indicated that collagen matrix could enhance the cell protective ability of EVs through inhibiting the apoptosis pathway and DNA damage.

### Collagen matrix improved proangiogenic capacity of EVs

**Fig. 5:**
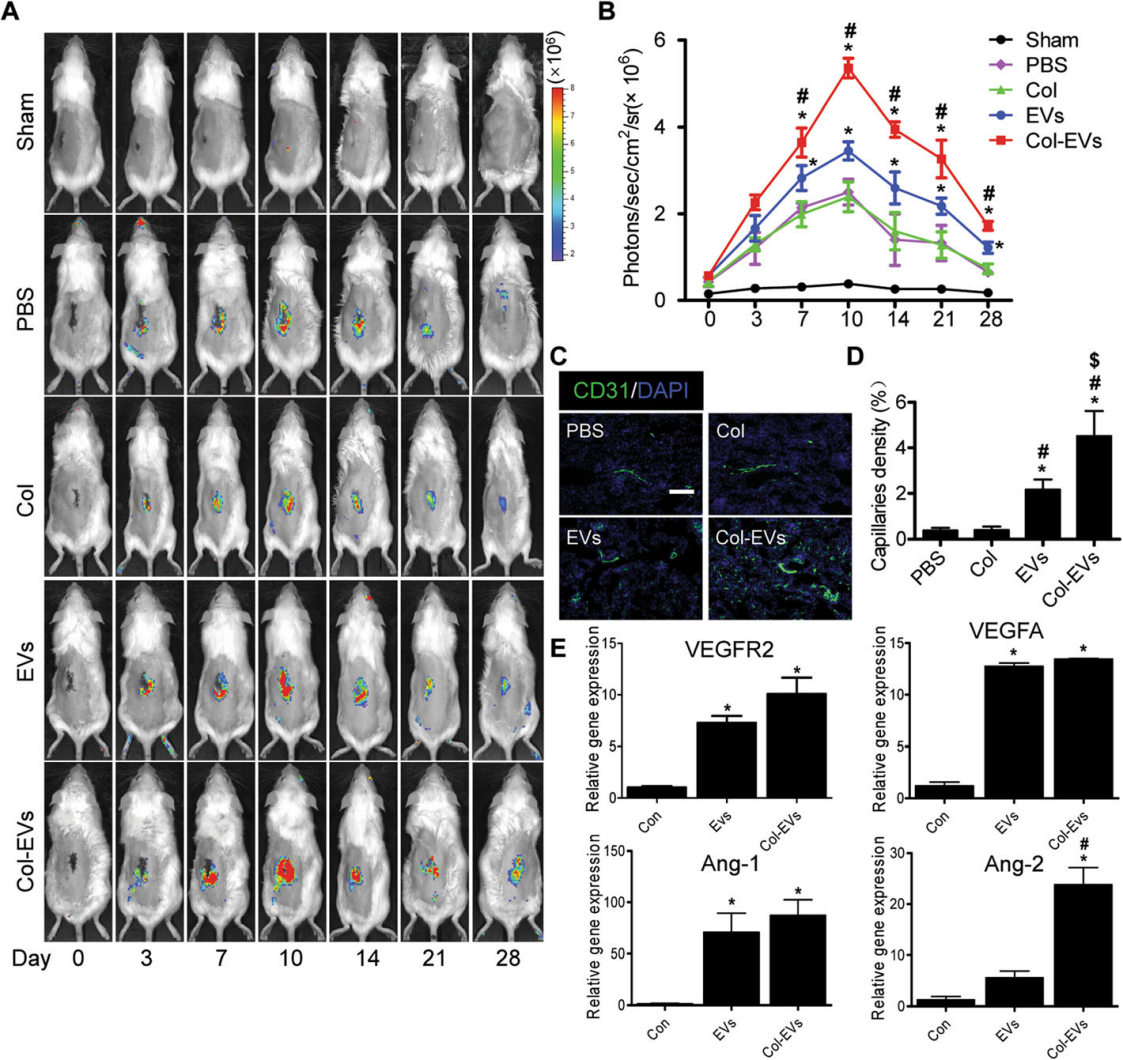
Collagen matrix application augmented the proangiogenic effects of EVs. **a** In vivo monitoring of the Vegfr2-luc expression following transplantation of Col-EVs or EVs by BLI in mouse AKI model. **b** Quantitative analysis of the dynamic trend of angiogenesis in ischemic kidneys via Fluc signals (*n* = 5, *P < 0.05 vs. PBS, ^#^*P* < 0.05 vs. EVs). **c** Representative images of kidney sections stained for CD31 (green) at day14. Scale bar, 100 μm. **d** Quantification of capillary density of ischemic kidneys in each group (*n* = 5; *P < 0.05 vs. PBS; ^#^*P* < 0.05 vs. Col; ^$^*P* < 0.05 vs. EVs). **e** Real-time qPCR analysis of angiogenic factor expression in HUVECs treated with 100 μg/mL EVs or Col-EVs for 24 h. Data were expressed as the mean ± SEM. *P < 0.05 vs. Con; ^#^*P* < 0.05 vs. EVs

To investigate whether collagen matrix gel could enhance the proangiogenic activity of EVs in vivo, EVs were transplanted into Vegfr2-luc transgenic mice which were subjected to kidney ischemia reperfusion injury (IRI). The Fluc signals were found in all groups other than sham, and the peak signal was detected in the Col-EVs group. The BLI signal observed in the PBS group indicates that injury could also initiate angiogenesis (Fig. 5a, b). Besides, immunostaining of CD31 revealed that microvascular density was significantly increased by Col-EVs application at 14 days, which was consistent with BLI data (Fig. 5c, d). These results suggested that the therapy based on Col-EVs dramatically increased the angiogenesis in the post-IR kidney.

To find out the underlying mechanisms of EV-induced angiogenesis, RT-PCR analysis was carried out to examine the expressions of proangiogenesis-related genes in the HUVECs. We found that the EVs and Col-EVs increased the expressions of proangiogenic genes Ang-1, Ang-2, VEGFA, and VEGFR2 in the endothelial cells (Fig. 5e). Together, these data indicated that collagen matrix could improve the proangiogenic activities of EVs and promote the kidney angiogenesis after injury.

### Renal recovery post AKI

**Fig. 6:**
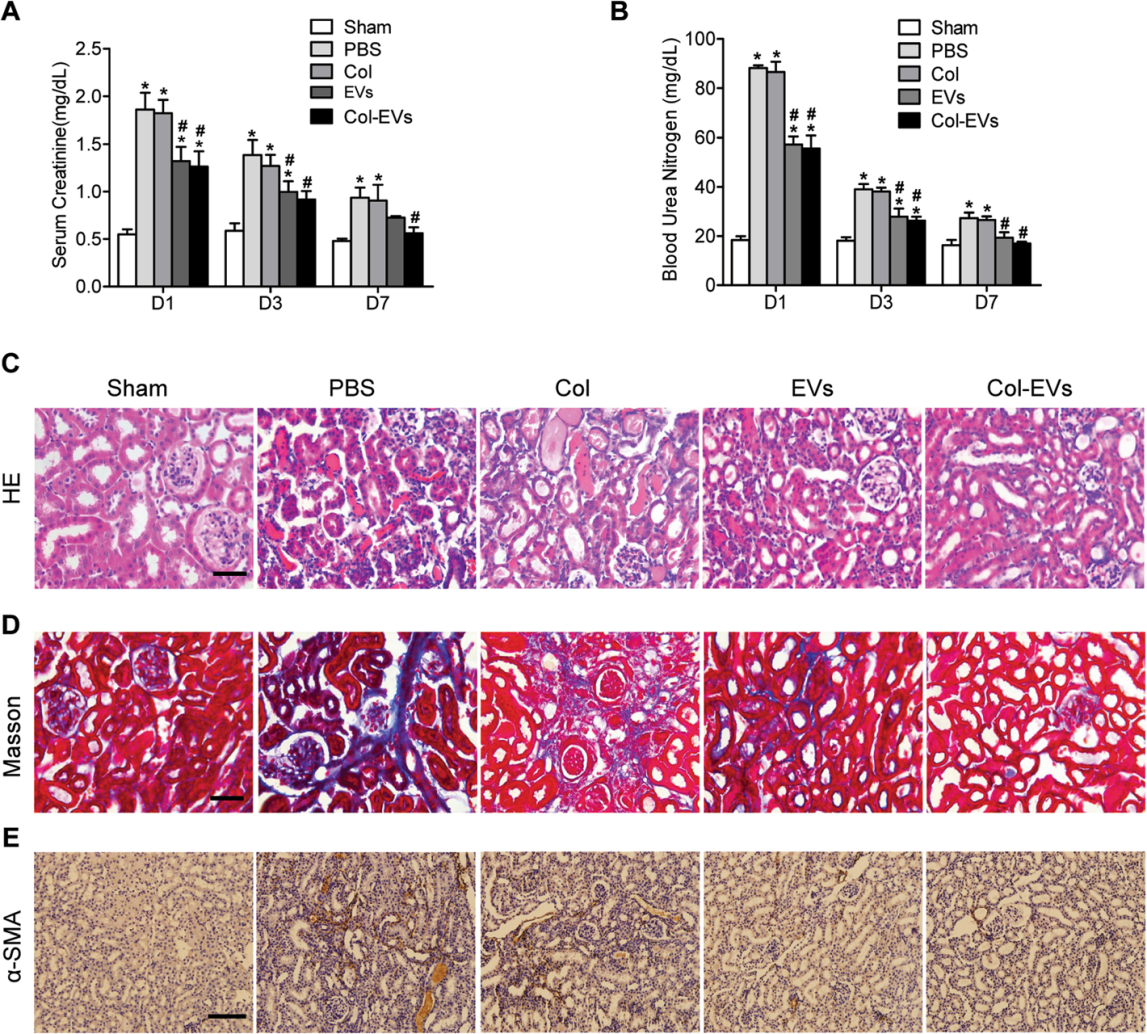
Treatment of Col-EVs accelerated renal recovery. **a** Serum creatinine and **b** BUN levels were measured at different time points after severe AKI (*n* = 5. *P < 0.05 vs. sham; ^#^*P* < 0.05 vs. PBS). **c** Representative images of kidney sections stained with HE at day 3 after AKI. Scale bar, 50 μm. **d** Masson trichrome staining and α-SMA staining (**e**) were performed to describe collagen deposition 1 month after AKI. Scale bar, 50 μm and 100 μm, respectively

To explore whether Col-EVs treatment facilitates kidney functional recovery, BUN and serum creatinine were examined. The renal function of AKI in mice significantly deteriorated, which was reflected in an increase in BUN and serum creatinine levels. Compared with the PBS group, the application of Col-EVs can markedly ameliorate renal function, which is manifested by a decrease in BUN and serum creatinine (Fig. 6a, b). Col-EV treatment demonstrated that necrotic tubules and hyaline casts were significantly relieved compared with the Col and PBS groups, indicating more evidently renoprotective actions of Col-EVs treatment (Fig. 6c, Figure S5 A, B). Masson trichrome staining showed an obvious reduction in the fibrotic area of the Col-EV group compared with other groups (Fig. 6d, Figure S5 C). The kidney sections stained with α-SMA demonstrated findings similar to those of Masson trichrome staining (Fig. 6e, Figure S5 D). These data revealed that Col-EVs have enormous anti-fibrotic effects in AKI.

### Inhibition of endoplasmic reticulum (ER) stress

**Fig. 7:**
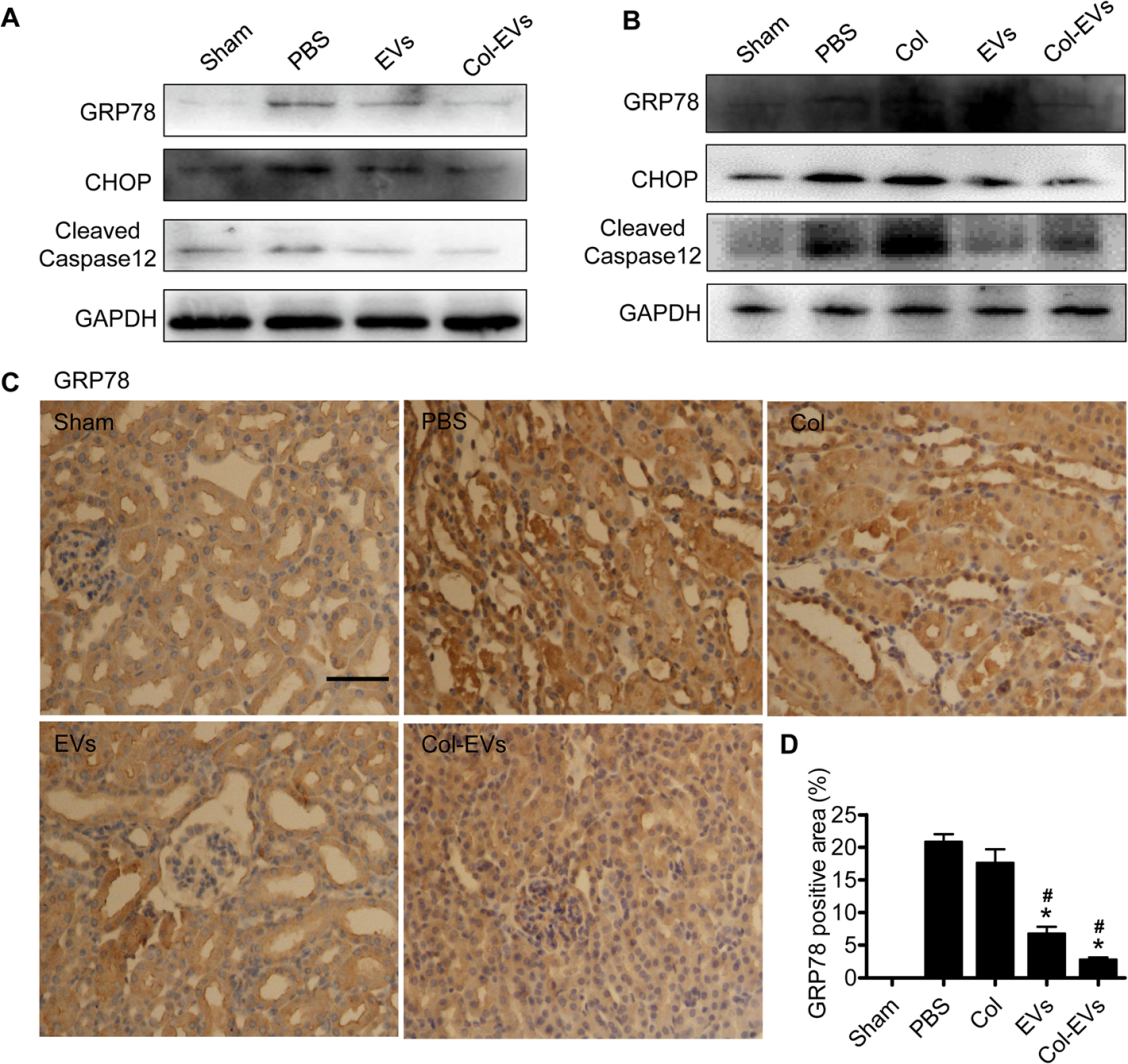
EVs derived from hP-MSCs protected injured kidneys through the suppression of IR-induced ER stress in vitro and in vivo. **a** The expression levels of ER stress-related proteins were detected at various groups in vitro using western blot. **b** The expression levels of ER stress-related proteins were detected in vivo using western blot. **c** Immunohistochemical staining of GRP78 three days after AKI. Scale bar, 100 μm. **d** Quantification of GRP78 staining area in each group. Data were represented as mean ± SEM (*n* = 5; *P < 0.05 vs. PBS; ^#^*P* < 0.05 vs. Col)

ER stress is considered to be a crucial mechanism for regulating cell damage and is involved in the process of I/R injury. We found that the expression of ER stress-related proteins, such as GRP78, was significantly increased in the PBS group, while those in EV and Col-EV group decreased (Fig. 7a). Impressively, similar results were yielded by western blot experiments in vivo (Fig. 7b). In addition, we further performed GRP78 immunohistochemical staining to evaluate the levels of ER stress (Fig. 7c, d). These results indicate that ER stress is closely involved in the protection effect of EVs, and EVs have a significant inhibitory effect on IR-induced ER stress.

## Discussion

In the present study, our results revealed that EVs derived from hP-MSCs attenuated murine AKI through the inhibition of ER stress in a model of renal IRI. Firstly, we found that collagen matrix markedly enhanced the retention and stability of EVs in the ischemic kidney as confirmed by BLI. Secondly, compared with other groups, Col-EVs have superior functions, including pathological damage reduction, promotion of renal tubular epithelial cell proliferation, inhibition of renal cell apoptosis, and enhancing of angiogenesis as well as ameliorating fibrosis. Consequently, the therapeutics of Col-EVs exhibited beneficial effects on functional and structural recovery in a murine AKI model. Finally, we explored the mechanism of EVs therapy, and we concluded that EVs exert therapeutic effects by inhibiting ER stress.

MSCs, located within the stroma of the bone marrow, placenta, umbilical cord, and adipose tissue, are adult stem cells with self-renewal and multi-directional differentiation potentials [34, 35]. It has been proposed that the therapeutic effects of MSCs after transplantation are mainly caused by a paracrine manner and initiate repair by immunomodulation, proliferation and anti-apoptosis, and stimulation of neovascularization [36, 37]. EVs are the secretory components of MSCs, and they are important mediators of cell-to-cell communication, both in physiological and pathological processes. EVs contribute to intercellular communication via transferring specific components, such as proteins and RNAs [38–40]. In fact, the therapeutic role of MSC-derived EVs in myocardial infarction [41, 42], kidney injury [43], and ischemic hindlimb has been demonstrated [44].

Collagen matrixes are the most commonly used biomaterials in skin, connective tissue, and peripheral nerve tissue engineering [45, 46]. In addition to their physiological properties as natural extracellular matrix components, their microstructure and stability also play a key role in cell-cell and cell-matrix interactions [33]. At the same time, as an active matrix material, collagen matrix is biodegradable to avoid long-term interference, but allows the tissue to be rapidly regenerated at a controlled rate [33, 47]. Previous studies have reported that collagen matrix is a good cell or drug delivery vehicle [48], so we hypothesized that the retention rate of EVs can be enhanced to further improve the therapeutic effects by binding to the collagen matrix. As the collagen gel biodegrades, its encapsulated EVs are slowly released, prolonging the residence time and sustained release. The collagen matrix has low immunogenicity, thereby establishing an immune-isolation barrier that protects the EVs from being cleared by the host immune system [11]. All in all, our strategy provides an effective way to optimize EV-induced tissue protection by using collagen matrix gel to encapsulate EVs to increase exogenous retention and stability for better therapeutic effects.

Emerging evidence strongly suggests that ER stress may be a potential mechanism for disease therapies and homeostatic regulation under stress, including I/R injury [39]. Using the renal I/R model, we discovered that increased ER stress leads to more apoptosis and damage; inhibition of ER stress enhances EV-induced protection against renal I/R injury. A series of studies have shown that the response to increasing unfolded and misfolded proteins causes GRP78 to dissociate from the ER stress sensor, while free GRP78 levels determine the activation of UPR, which may trigger cell death under ER stress [49–52]. Therefore, the regulation of GRP78 has a close impact on the ER stress process. Nevertheless, the contents in the EVs regulate the expression of GRP78 remains to be resolved.

## Conclusion

In conclusion, collagen matrix was used for the first time to encapsulate EVs to achieve sustained release in the kidney, so as to achieve better therapeutic effect. Encapsulating EVs with this biodegradable collagen matrix could remarkably augment their retention and stability at the injured sites. Col-EVs notably improved the proliferation, anti-apoptosis, and angiogenesis of tubular epithelial cells, accelerating the recovery of AKI. Moreover, the BLI technology used in this study allows us to non-invasive track the administered EVs in vivo in real-time, which may significantly promote EV research and applications. In summary, the strategy depicted in this study opened the door to new treatment for patients with AKI and had great practical value.

## Supplementary information

**Additional file 1.**

## Data Availability

All data generated and/or analyzed during this study are available from the corresponding author upon reasonable request.

## Abbreviations

AKI: Acute kidney injury
BLI: Bioluminescence imaging
BUN: Blood urea nitrogen
CKD: Chronic kidney disease
ECM: Extracellular matrix
EVs: Extracellular vesicles
GFP: Green fluorescent protein
Gluc: *Gaussia* luciferase
hP-MSCs: Human placental mesenchymal stem cell
I/R: Ischemia reperfusion
IRI: Ischemia reperfusion injury
MSCs: Mesenchymal stem cells
SCr: Serum creatinine
SEM: Scanning electron microscopy

## References

[1] Xu L, Li X, Zhang F, Wu L, Dong Z, Zhang D (2019). EGFR drives the progression of AKI to CKD through HIPK2 overexpression. Theranostics..

[2] Feng G, Zhang J, Li Y, Nie Y, Zhu D, Wang R (2016). IGF-1 C domain-modified hydrogel enhances cell therapy for AKI. J Am Soc Nephrol.

[3] Zhu F, OLS CLS, Pei G, Hu Z, Yang J, Zhu H (2017). Adipose-derived mesenchymal stem cells employed exosomes to attenuate AKI-CKD transition through tubular epithelial cell dependent Sox9 activation. Oncotarget..

[4] Liu N, Patzak A, Zhang J (2013). CXCR4-overexpressing bone marrow-derived mesenchymal stem cells improve repair of acute kidney injury. Am J Physiol Renal Physiol.

[5] Little MH (2006). Regrow or repair: potential regenerative therapies for the kidney. J Am Soc Nephrol.

[6] Kaushal GP, Shah SV (2014). Challenges and advances in the treatment of AKI. J Am Soc Nephrol.

[7] Jia XH, Lu H, Li C, Feng GW, Yao XP, Mao LN (2013). Human embryonic stem cells-derived endothelial cell therapy facilitates kidney regeneration by stimulating renal resident stem cell proliferation in acute kidney injury. Chin Sci Bull.

[8] Feng G, Mao D, Che Y, Su W, Wang Y, Xu Y (2013). The phenotypic fate of bone marrow-derived stem cells in acute kidney injury. Cell Physiol Biochem.

[9] Collino F, Bruno S, Incarnato D, Dettori D, Neri F, Provero P (2015). AKI recovery induced by mesenchymal stromal cell-derived extracellular vesicles carrying microRNAs. J Am Soc Nephrol.

[10] Mae SI, Shono A, Shiota F, Yasuno T, Kajiwara M, Gotoda-Nishimura N (2013). Monitoring and robust induction of nephrogenic intermediate mesoderm from human pluripotent stem cells. Nat Commun.

[11] Zhang K, Zhao X, Chen X, Wei Y, Du W, Wang Y (2018). Enhanced therapeutic effects of mesenchymal stem cell-derived exosomes with an injectable hydrogel for hindlimb ischemia treatment. ACS Appl Mater Interfaces.

[12] Zhang ZG, Buller B, Chopp M (2019). Exosomes - beyond stem cells for restorative therapy in stroke and neurological injury. Nat Rev Neurol.

[13] Imai T, Takahashi Y, Nishikawa M, Kato K, Morishita M, Yamashita T (2015). Macrophage-dependent clearance of systemically administered B16BL6-derived exosomes from the blood circulation in mice. J Extracell Vesicles.

[14] Subra C, Laulagnier K, Perret B, Record M (2007). Exosome lipidomics unravels lipid sorting at the level of multivesicular bodies. Biochimie..

[15] Abello J, Nguyen TDT, Marasini R, Aryal S, Weiss ML (2019). Biodistribution of gadolinium- and near infrared-labeled human umbilical cord mesenchymal stromal cell-derived exosomes in tumor bearing mice. Theranostics..

[16] Berthod F, Saintigny G, Chretien F, Hayek D, Collombel C, Damour O (1994). Optimization of thickness, pore size and mechanical properties of a biomaterial designed for deep burn coverage. Clin Mater.

[17] Zhao X, Cui K, Li Z (2019). The role of biomaterials in stem cell-based regenerative medicine. Future Med Chem.

[18] Sun J, Mou C, Shi Q, Chen B, Hou X, Zhang W (2018). Controlled release of collagen-binding SDF-1alpha from the collagen scaffold promoted tendon regeneration in a rat Achilles tendon defect model. Biomaterials..

[19] Zhang K, Jia Z, Yang B, Feng Q, Xu X, Yuan W (2018). Adaptable hydrogels mediate cofactor-assisted activation of biomarker-responsive drug delivery via positive feedback for enhanced tissue regeneration. Adv Sci (Weinh).

[20] Wang M, Wang C, Chen M, Xi Y, Cheng W, Mao C (2019). Efficient angiogenesis-based diabetic wound healing/skin reconstruction through bioactive antibacterial adhesive ultraviolet shielding nanodressing with exosome release. ACS Nano.

[21] Peppas NA, Hilt JZ, Khademhosseini A, Langer R (2006). Hydrogels in biology and medicine: from molecular principles to bionanotechnology. Adv Mater.

[22] Liu B, Lee BW, Nakanishi K, Villasante A, Williamson R, Metz J (2018). Cardiac recovery via extended cell-free delivery of extracellular vesicles secreted by cardiomyocytes derived from induced pluripotent stem cells. Nat Biomed Eng.

[23] Liu X, Yang Y, Li Y, Niu X, Zhao B, Wang Y (2017). Integration of stem cell-derived exosomes with in situ hydrogel glue as a promising tissue patch for articular cartilage regeneration. Nanoscale..

[24] Shi Q, Qian Z, Liu D, Sun J, Wang X, Liu H (2017). GMSC-derived exosomes combined with a chitosan/silk hydrogel sponge accelerates wound healing in a diabetic rat skin defect model. Front Physiol.

[25] Xu N, Wang L, Guan J, Tang C, He N, Zhang W (2018). Wound healing effects of a Curcuma zedoaria polysaccharide with platelet-rich plasma exosomes assembled on chitosan/silk hydrogel sponge in a diabetic rat model. Int J Biol Macromol.

[26] Zhang Y, Xu J, Liu S, Lim M, Zhao S, Cui K (2019). Embryonic stem cell-derived extracellular vesicles enhance the therapeutic effect of mesenchymal stem cells. Theranostics..

[27] Tao H, Chen X, Wei A, Song X, Wang W, Liang L, et al. Comparison of teratoma formation between embryonic stem cells and parthenogenetic embryonic stem cells by molecular imaging. Stem Cell Int. 2018;2018:7906531. 10.1155/2018/7906531 PMC5889892 29765423

[28] Zhao N, Yue Z, Cui J, Yao Y, Song X, Cui B (2019). IGF-1C domain-modified hydrogel enhances therapeutic potential of mesenchymal stem cells for hindlimb ischemia. Stem Cell Res Ther.

[29] Chen CC, Liu L, Ma F, Wong CW, Guo XE, Chacko JV (2016). Elucidation of exosome migration across the blood-brain barrier model in vitro. Cell Mol Bioeng.

[30] Zhang S, Liu Y, Zhang X, Zhu D, Qi X, Cao X (2018). Prostaglandin E2 hydrogel improves cutaneous wound healing via M2 macrophages polarization. Theranostics..

[31] Hoshino A, Costa-Silva B, Shen TL, Rodrigues G, Hashimoto A, Tesic Mark M (2015). Tumour exosome integrins determine organotropic metastasis. Nature..

[32] Sharfuddin AA, Molitoris BA (2011). Pathophysiology of ischemic acute kidney injury. Nat Rev Nephrol.

[33] Noah EM, Chen J, Jiao X, Heschel I, Pallua N (2002). Impact of sterilization on the porous design and cell behavior in collagen sponges prepared for tissue engineering. Biomaterials..

[34] Huang A, Liu D, Qi X, Yue Z, Cao H, Zhang K (2019). Self-assembled GFFYK peptide hydrogel enhances the therapeutic efficacy of mesenchymal stem cells in a mouse hindlimb ischemia model. Acta Biomater.

[35] Tao H, Han Z, Han ZC, Li Z (2016). Proangiogenic features of mesenchymal stem cells and their therapeutic applications. Stem Cells Int.

[36] Togel FE, Westenfelder C (2012). Kidney protection and regeneration following acute injury: progress through stem cell therapy. Am J Kidney Dis.

[37] Togel FE, Westenfelder C (2010). Mesenchymal stem cells: a new therapeutic tool for AKI. Nat Rev Nephrol..

[38] Bei Y, Das S, Rodosthenous RS, Holvoet P, Vanhaverbeke M, Monteiro MC (2017). Extracellular vesicles in cardiovascular theranostics. Theranostics..

[39] Liao Z, Luo R, Li G, Song Y, Zhan S, Zhao K (2019). Exosomes from mesenchymal stem cells modulate endoplasmic reticulum stress to protect against nucleus pulposus cell death and ameliorate intervertebral disc degeneration in vivo. Theranostics..

[40] Wang C, Wang M, Xu T, Zhang X, Lin C, Gao W (2019). Engineering bioactive self-healing antibacterial exosomes hydrogel for promoting chronic diabetic wound healing and complete skin regeneration. Theranostics..

[41] Lai RC, Arslan F, Lee MM, Sze NS, Choo A, Chen TS (2010). Exosome secreted by MSC reduces myocardial ischemia/reperfusion injury. Stem Cell Res.

[42] Waldenstrom A, Ronquist G (2014). Role of exosomes in myocardial remodeling. Circ Res.

[43] Eirin A, Zhu XY, Puranik AS, Tang H, McGurren KA, van Wijnen AJ (2017). Mesenchymal stem cell-derived extracellular vesicles attenuate kidney inflammation. Kidney Int.

[44] Du W, Zhang K, Zhang S, Wang R, Nie Y, Tao H (2017). Enhanced proangiogenic potential of mesenchymal stem cell-derived exosomes stimulated by a nitric oxide releasing polymer. Biomaterials..

[45] Sheridan RL, Tompkins RG (1999). Skin substitutes in burns. Burns..

[46] Friess W (1998). Collagen--biomaterial for drug delivery. Eur J Pharm Biopharm.

[47] Langer R, Vacanti JP (1993). Tissue engineering. Science..

[48] Wang N, Xiao Z, Zhao Y, Wang B, Li X, Li J (2018). Collagen scaffold combined with human umbilical cord-derived mesenchymal stem cells promote functional recovery after scar resection in rats with chronic spinal cord injury. J Tissue Eng Regen Med.

[49] Wang C, Zhu G, He W, Yin H, Lin F, Gou X (2019). BMSCs protect against renal ischemia-reperfusion injury by secreting exosomes loaded with miR-199a-5p that target BIP to inhibit endoplasmic reticulum stress at the very early reperfusion stages. FASEB J.

[50] Corazzari M, Gagliardi M, Fimia GM, Piacentini M (2017). Endoplasmic reticulum stress, unfolded protein response, and cancer cell fate. Front Oncol.

[51] Mann MJ, Hendershot LM (2006). UPR activation alters chemosensitivity of tumor cells. Cancer Biol Ther.

[52] Taniguchi M, Yoshida H (2015). Endoplasmic reticulum stress in kidney function and disease. Curr Opin Nephrol Hypertens.

