# Supplementary material for: Enhanced therapeutic effects of MSC-derived extracellular vesicles with an injectable collagen matrix for experimental acute kidney injury treatment
Source: Stem Cell Res Ther. 2020 Apr 22;11:161. doi: 10.1186/s13287-020-01668-w (PMC7178991; doi:10.1186/s13287-020-01668-w)
Supplement: Supplementary file 1 — Additional file 1. [file 13287_2020_1668_MOESM1_ESM.docx]

**SUPPLEMENTAL DATA**

**Supplementary Methods**

**Cell culture**

Human placenta-derived MSCs (hP-MSCs) were isolated and cultured as reported previously.([1](#_ENREF_1), [2](#_ENREF_2)) In generally, we cultured hP-MSCs in DMEM/F12 (Gibco, Grand Island, NY) complete medium containing 10% fetal bovine serum (FBS, HyClone, Austrial) and 1% penicillin-streptomycin (Gibco). To obtain EV-free FBS, we performed ultracentrifugation at 100000 g for 70 min.([3](#_ENREF_3)) We used hP-MSCs which passages between 4 and 8 for subsequent experiments. Human renal proximal tubular (HK-2) cells and human umbilical vein endothelial cells (HUVECs) were purchased from ATCC (ATCC, Manassas, VA). The HK-2/HUVECs were cultured in DMEM (Gibco) and EGM-2 (Lonza, Walkersville, MD) respectively, with the following components, 10% FBS, 1% penicillinand streptomycin and 1% L-glutamine. All cells were cultured at 37°C in a humidified 5% CO_2_, 95% air incubator.

**EV characterization**

Transmission electron microscopy (TEM; Talos F200C, Thermo Fisher, MA) was performed to visualize and examine the morphology of EVs. The samples were deposited on copper grids covered with carbon film (Zhongjingkeyi Technology, Beijing, China) and dried at room temperature for 2 min. The excess liquid was removed with a filter, and the samples were negatively stained with 2% uranyl acetate for 30 s. The samples were air dried for 60 min and then imaged using TEM. The size and concentration of EVs were determined using nanoparticle tracking analysis (NTA) (Particle Metrix, Germany). The test parameters were set with 100 nm polystyrene-latex beads as standards. EVs were diluted 4000- and 2000- fold, respectively, using distilled deionize water, to obtain 20 and 100 objects per frame. Each sample was measured in triplicate at the camera setting with an acquisition time of 60 s. The marker proteins of EVs for Western blot analysis are CD9 (1:1000, Abcam, Cambridge, MA), ALIX (1: 1000, Wanleibio, Shenyang, China) and TSG101 (1:1000, Abcam, Cambridge, MA).

**EVs Internalization**

On the one hand, we extracted Gluc-labeled EVs from hP-MSCs expressing Gluc-lactadherin fusion proteins and suspended in PBS.([3](#_ENREF_3)) We inoculated HK-2 into a 24-well plate at a seeding rate of 5×10^4^ cells per well and Gluc-labeled EVs (100 μg/ml) were added to the medium after 24 h, then incubated for 0.5, 1, 3, 6 and 12 h, respectively. After washing twice with PBS, the coelenterazine (Nanolight technology, Pinetop, AZ) substrate was added and the Gluc-EVs internalized into the cells were analyzed using IVIS Lumina Imaging System (Xenogen Corporation, Hopkinto, MA). On the other hand, EVs were labeled with the CM-DiI membrane dye (Invitrogen, Carlsbad, CA) according to the manufacturer's instructions. Briefly, EVs were mixed with 1 μmol/L CM-DiI, and incubated for 10 min at 37 °C, and then the unbound dye was removed by ultracentrifugation at 100000 g for 70 min at 4 °C. The resulting EVs pellets were resuspended in PBS. The CM-DiI labeled EVs were incubated with HK-2 cells expressing green fluorescent protein (GFP) for 12 h at 37 °C. HK-2 cells were washed with PBS and fixed in 4% paraformaldehyde, and the uptake of EVs by cells was observed by fluorescence microscopy.

**Scratch wound healing assay**

To assess the ability of Col-EVs to promote cell migration, we performed a scratch wound healing test. HK-2 cells were seeded into a 24-well plate with complete DMEM/F12 media. When cells reached 90% confluence, scratch wounds were generated with a sterile plastic 10 µl-micropipette tip, then added EVs, Col-EVs or PBS into the plate. With an inverted microscopy (Olympus, Lake Success, NY), images of at least five fields of view were taken for each scratch at 0, 18, and 36 h, and then the migration distance was quantified using Image J software.

**Quantitative real time-PCR**

Total RNA was extracted from the cells using Trizol reagent (Invitrogen, Grand Island, NY) according to the manufacturer's manuals, and 1 μg of RNA was reverse transcribed into cDNA using the First-strand cDNA Synthesis System (TransGen Biotech, Beijing, China). All Quantitative PCR experiments were performed on a CFX96TM real-time PCR machine using SYBR master-mix (Qiagen, Germany) and gene-specific primers. The relative expression level of the mRNA was calculated using 2-ΔΔCt method and normalized to GAPDH. The primer sequences used in this study are shown in **Suppl. Table 1**.

**Western blotting analysis**

Tissues and HK-2 cells were lysed with RIPA buffer (Solarbio, Shanghai, China) with PMSF (Roche Molecular BioChemicals, Indianapolis, IN). The protein concentrations were quantified by BCA protein assay Kit (Promega, Madison, WI). The total proteins (30 μg) were separated by 12% SDS-PAGE and transferred to polyvinylidene fluoride (PVDF; Millipore, Watford, UK) membranes. The membranes were blocked with 5% skim milk for 2 h, then incubated overnight in the primary antibodies at 4°C, then incubated for 2 h at room temperature with the appropriate secondary antibodies at the next day. After that the protein signal was measured using the West Pico Chemiluminescent Substrate Kit (Pierce, Rockford, IL). The following primary antibodies were used: GRP78 (1:1000, Wanleibio, China), CHOP (1:1000, CellSignalingTech, USA), Cleaved- Caspase12 (1:500, Wanleibio, China) and GAPDH (1:1000, Beyotime, China). Signals of Western blot was detected using Luminata crescendo western horseradish peroxidase substrate (Millipore).

**Preparation of collagen matrix**

In this study, we used rat tail collagen I (3 mg/mL) purchased from Corning corporation. First, we placed corning collagen I, rat tail, sterile 10× PBS, ddH_2_O and sterile 1 N NaOH on ice. Next, we determined the final volume of Collagen I solution to be used and the desired final collagen concentration (1.5 mg/ml). Then following the manufacturer's instructions, we add suitable volumes of 10×PBS, 1 N NaOH, ddH_2_O in order. Finally, adding a calculated volume of collagen I solution and mix it so that the final pH is around 7 (the pH value needs to be determined for initial use). In order to obtain collagen matrix-incorporated EVs, 100 μg of EVs were mixed with collagen matrix to finally obtain a working solution of Col-EVs. After incubation at 37°C for 30 min, the Col-EVs solution can be crosslinked into the gel.

**Histochemical and immunofluorescence staining**

The kidneys were fixed with 4% paraformaldehyde for 24 h, embedded in paraffin, and sectioned (5-8 µm). Hematoxylin eosin (HE) staining was used to estimate pathological injury, and Masson staining was conducted to evaluate the extent of kidney fibrosis. For immunofluorescence and immunohistochemistry analysis, kidney tissues were embedded in OCT (Sakura Finetek, Tokyo, Japan), snap frozen with dry ice, and cut into 6 μm thick sections. The slides or cells were incubated with primary antibodies against Ki67 (1;200, Abcam, UK), Cleaved- Caspase12 (1:200), CD31 (1:200, BD, USA), α-SMA (1:100, Wanleibio, Shenyang, China) and GRP78 (1:200) at 4°C overnight and further incubated with appropriate fluorescently labeled secondary antibodies the next day (Life Technologies, Carlsbad, CA). Nuclei were stained with DAPI. The fluorescent images were analyzed using ImageJ software.

**Supplemental Table**

Table S1 human primers used for RT-PCR

| Gene Name | Primers |
| --- | --- |
| GAPDH | Forward: GGAGCGAGATCCCTCCAAAAT  Reverse: GGCTGTTGTCATACTTCTCATGG |
| ANG-1 | Forward: CGCCGAAGTCCAGAAAACAG  Reverse: GGGAAGAGAAATCCGGTTCCA |
| ANG-2 | Forward: GCTCGAATACGATGACTCGGT  Reverse: GTTTGCTCCGCTGTTTGGTT |
| VEGFA | Forward: TGTCTAATGCCCTGGAGCCT  Reverse: GTCACATCTGCAAGTACGTTCG |
| VEGFR2 | Forward: CAAGTGGCTAAGGGCATGGA  Reverse: ATTTCAAAGGGAGGCGAGCA |
|  |  |

**Supplemental Figures & Legends**

**
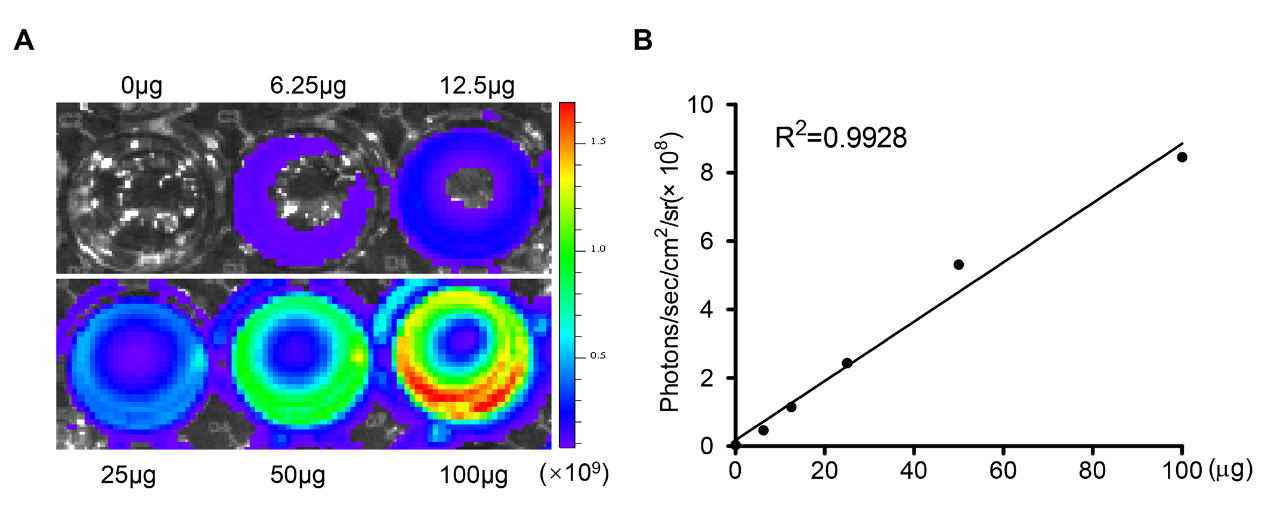
**

**Supplementary Figure 1. Bioluminescent labeling of EVs.** (**A-B**) Ex vivo imaging of Gluc-labeled EVs showed increasing bioluminescence signals with concentrations of EVs (R^2^ = 0.9892).


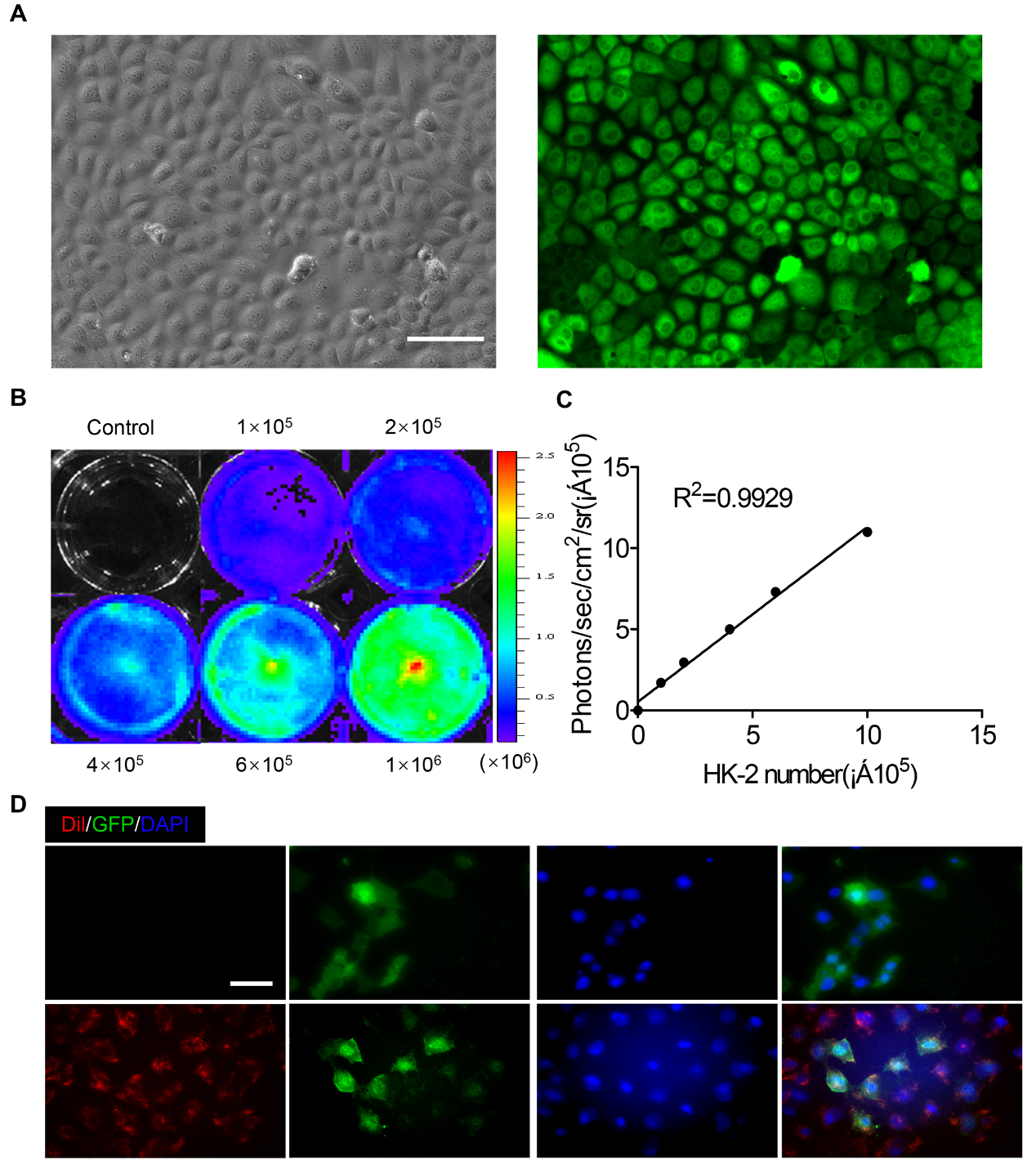


**Supplementary Figure 2. Characterization of HK-2 (GFP-Flu) and bioluminescent EVs (Gluc) and internalization of DiI-labeled EVs. (A)** HK-2 were GFP positive. Scale bar, 100 μm. (**B-C**) BLI quantification demonstrated a robust linear correlation between the number of HK-2 and Fluc average radiance. (**D**) HK-2 cells expressing green fluorescent protein (GFP, green) could take up CM-DiI-labeled EVs (red). Scale bar, 100 μm. All experiments were performed in triplicate.


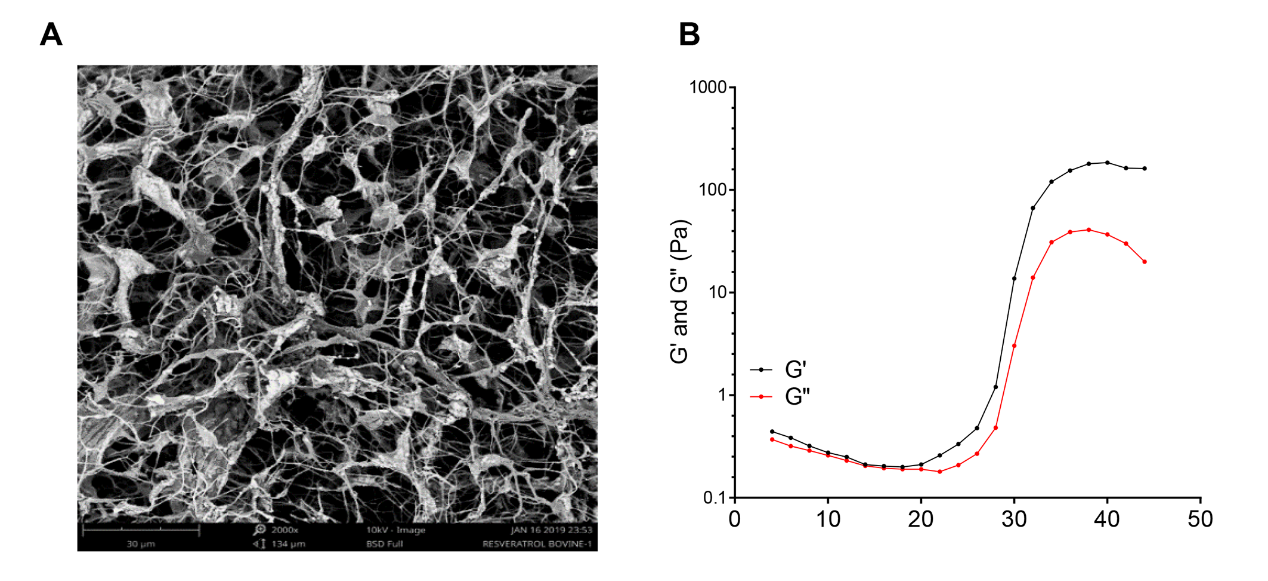


**Supplementary Figure 3. Characterization of thermosensitive and pH-responsive collagen matrix gel.** (**A**) Scanning electron microscopy image of collagen hydrogel. (**B**) The rheological properties of the collagen matrix gel as a function of temperature were evaluated. All experiments were performed in triplicate.


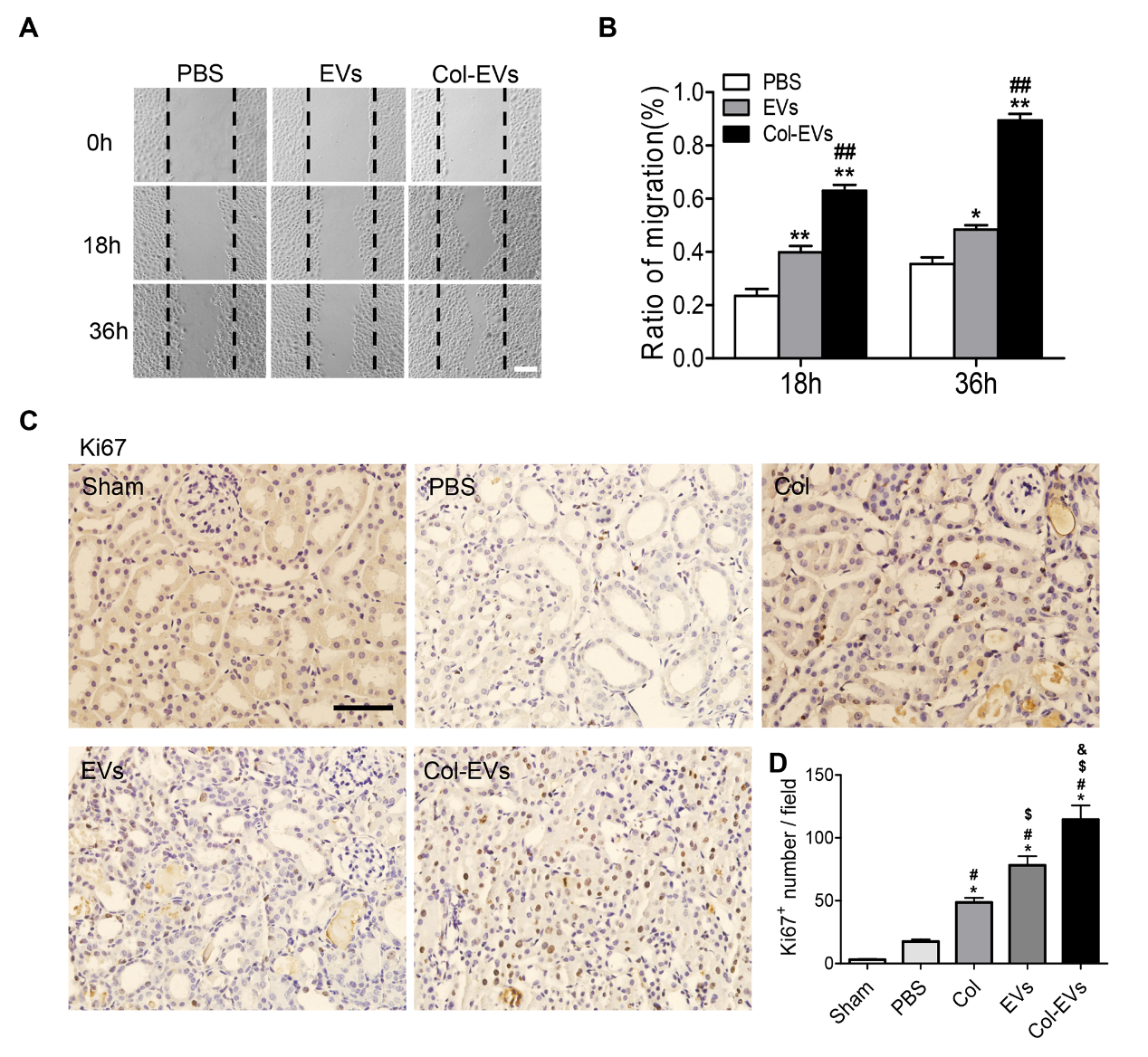


**Supplementary Figure 4. Enhanced proliferation effects were promoted by Col-EVs.** (**A**). Scratch wound-healing assay of HK-2 cells treated with EVs and Col-EVs. Scale bar, 100um. (**B**) Diagram of the ratio of cell migration. Data were expressed as the mean ± SEM. **P*<0.05 vs PBS, ^#^*P*<0.05 vs EVs. (**C**) Immunohistochemical staining of Ki67 at day 3 after AKI. Scale bar, 50um. (**D**) Quantification of Ki67+ cell numbers in each group. Data were represented as mean ± SEM. **P* <0.05 vs Sham; *^#^P*<0.05 vs PBS; ^$^*P*<0.05 vs Col; ^&^*P*<0.05 vs EVs. All experiments were performed in triplicate.

**
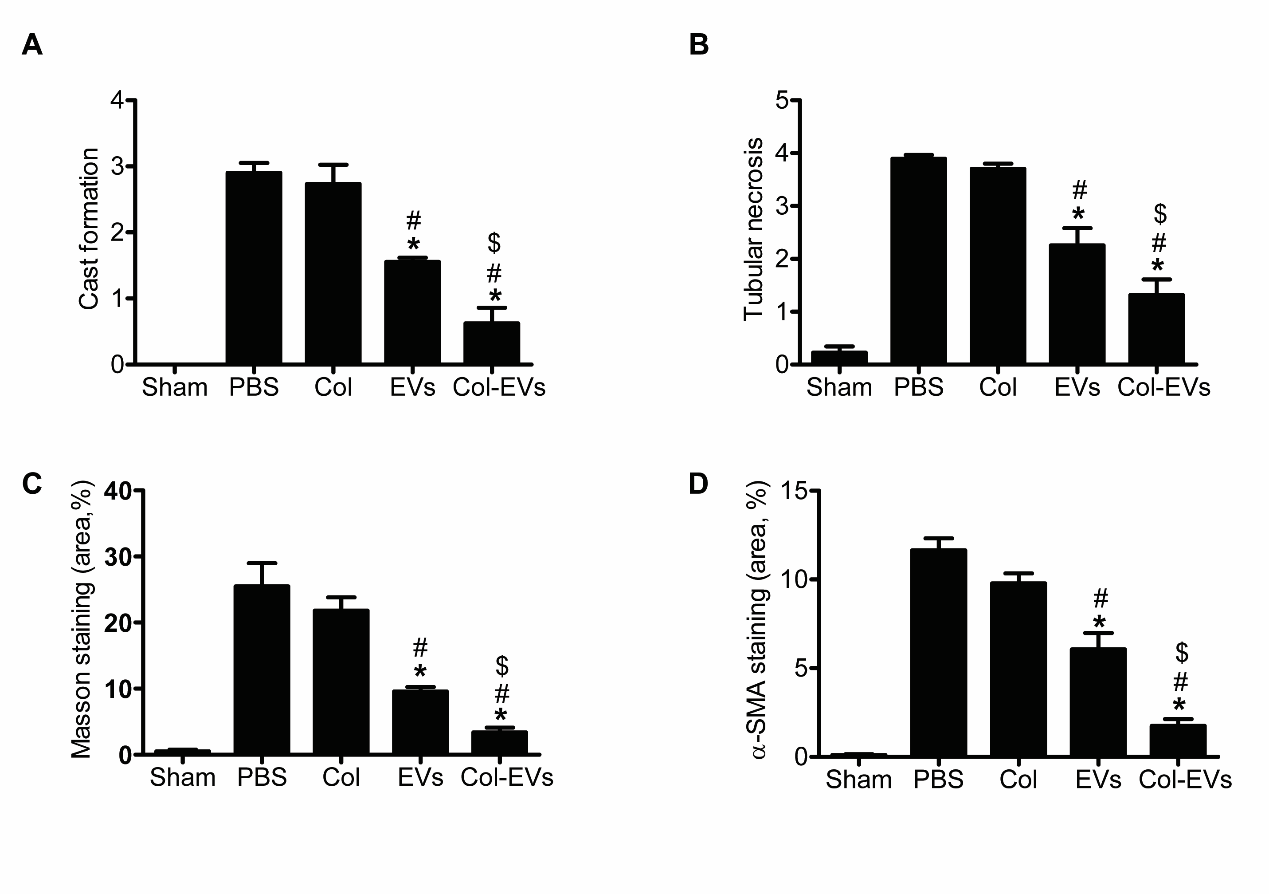
**

**Supplementary Figure 5. Quantitative statistical results of staining.** (**A**). Quantitative histologic assessment of hyaline cast formation. (**B**) Quantitative histologic assessment of tubular necrosis. (**C**) Quantification of Masson staining. (**D**) Quantification of α-SMA staining. Data were represented as mean ± SEM. (n=5; **P* <0.05 vs PBS; ^#^*P*<0.05 vs Col; ^$^*P*<0.05 vs EVs).

**REFERENCES**

1. Lu LL., Liu YJ., Yang SG., Zhao QJ., Wang X., Gong W., et al. (2006). Isolation and characterization of human umbilical cord mesenchymal stem cells with hematopoiesis-supportive function and other potentials. Haematologica, 91(8):1017-28.

2. Liang L., Li Z., Ma T., Han Z., Du W., Geng J., et al. (2017). Transplantation of Human Placenta-Derived Mesenchymal Stem Cells Alleviates Critical Limb Ischemia in Diabetic Nude Rats. Cell transplantation, 26(1):45-61.

3. Zhang K., Zhao X., Chen X., Wei Y., Du W., Wang Y., et al. (2018).Enhanced Therapeutic Effects of Mesenchymal Stem Cell-Derived Exosomes with an Injectable Hydrogel for Hindlimb Ischemia Treatment. ACS Appl Mater Interfaces, 10(36), 30081-91.
